# Titanium-Based Biomaterials for Preventing Stress Shielding between Implant Devices and Bone

**DOI:** 10.1155/2011/836587

**Published:** 2011-06-22

**Authors:** M. Niinomi, M. Nakai

**Affiliations:** Institute for Materials Research, Tohoku University, 2-1-1 Aoba-ku, Katahira, Sendai 980-8577, Japan

## Abstract

*β*-type titanium alloys with low Young's modulus are required to inhibit bone atrophy and enhance bone remodeling for implants used to substitute failed hard tissue. At the same time, these titanium alloys are required to have high static and dynamic strength. On the other hand, metallic biomaterials with variable Young's modulus are required to satisfy the needs of both patients and surgeons, namely, low and high Young's moduli, respectively. In this paper, we have discussed effective methods to improve the static and dynamic strength while maintaining low Young's modulus for *β*-type titanium alloys used in biomedical applications. Then, the advantage of low Young's modulus of *β*-type titanium alloys in biomedical applications has been discussed from the perspective of inhibiting bone atrophy and enhancing bone remodeling. Further, we have discussed the development of *β*-type titanium alloys with a self-adjusting Young's modulus for use in removable implants.

## 1. Introduction

It is well known that the stress transfer between an implant device and a bone is not homogeneous when Young's moduli of the implant device and the bone are different; this is defined as stress shielding. In such conditions, bone atrophy occurs and leads to the loosening of the implant and refracturing of the bone [1]. Therefore, it is desirable if the stiffness (Young's modulus) is not too high compared to that of bone. Implant devices are mainly made from metallic biomaterials such as stainless steels, Co-Cr alloys, and titanium (Ti) and its alloys. Young's moduli of these metallic biomaterials are generally much greater than that of the bone. Young's moduli of the most widely used stainless steel for implant devices, SUS316L stainless steel and Co-Cr alloys, are around 180 GPa and 210 GPa, respectively [2]. Young's moduli of Ti (pure titanium) and its alloys are generally smaller than those of stainless steels and Co-Cr alloys. For example, Ti and its alloy, Ti-6Al-4V ELI, which are widely used for constructing implant devices, have a Young's modulus of around 110 GPa. However, this value is still higher than that of the bone, that is, 10–30 GPa [3]. 

Ti alloys are grouped into *α*-, (*α* + *β*)-, and *β*-type alloys. Young's moduli of *α*- and (*α* + *β*)-type titanium alloys such as Ti and Ti-6Al-4V ELI are higher than those of *β*-type titanium alloys. Therefore, *β*-type titanium alloys are advantageous for the development of titanium alloys with low Young's modulus for biomedical applications. Researchers have been focusing on reducing Young's moduli of *β*-type titanium alloys for use in biomedical applications because they are composed of toxicity- and allergy-free elements. A number of *β*-type titanium alloys mainly composed of toxicity- and allergy-free elements and with low Young's moduli have been developed or are still being developed [4]. Their Young's moduli are approximately below 80 GPa in solution-treated conditions. Young's modulus of a material can be different depending on the type of measurement methods used, such as tensile tests, three-point bending tests, and free resonance methods. The lowest value of Young's modulus reported for the polycrystal *β*-type titanium alloy, Ti-35Nb-4Sn [5], or Ti-24Nb-4Zr-7.9Sn [6], subjected to severe cold working, is around 40 GPa. 

The authors have also developed a *β*-type titanium alloy, Ti-29Nb-13Ta-4.6Zr, referred to as TNTZ, that is composed of toxicity- and allergy-free elements and that has a low Young's modulus. Young's modulus of TNTZ subjected to solution treatment and measured by a resonance method has been found to be around 60 GPa [7]. This value is lowered to around 55 GPa by severe working such as severe cold rolling and cold swaging [8]. 

The strength as well as Young's modulus of titanium alloys is a very important factor for their long-term use in implants for biomedical applications. In particular, dynamic strength such as fatigue strength is highly important. Developing the fatigue strength and simultaneously lowering Young's modulus is somewhat difficult because they are opposite natures when the bonding force between atoms is considered. The fatigue strength of *β*-type titanium alloys used in biomedical applications with maintaining the Young's modulus as low as possible is currently being developed [9].

Concerning Young's modulus, the level of the value of Young's modulus, which is effective to prevent the stress shielding between the implant made of the low-Young's modulus *β*-type titanium alloy and bone should be proved. The effect of Young's modulus on bone atrophy has previously been investigated using implants made of titanium alloys with different Young's moduli [8].

This paper chiefly describes the simultaneous improvement of the dynamic strength and lowering Young's modulus of the *β*-type titanium alloy, TNTZ. The effect of Young's modulus on bone atrophy and bone remodeling has also been discussed in this paper.

## 2. Improvement of Static Strength while Maintaining Low Young's Modulus

Improving static strength such as tensile strength can be achieved by employing strengthening mechanisms such as work hardening, grain refinement strengthening, precipitation strengthening, and dispersion strengthening. One of the best ways to increase tensile strength while maintaining a Young's modulus low is to generally introduce many dislocations by ordinal severe cold working such as severe cold rolling and swaging and special severe cold working such as high pressure torsion (HPT), accumulative roll-bonding (ARB), and equal channel angular pressing (ECAP) [10]. 

**Figure 1:**
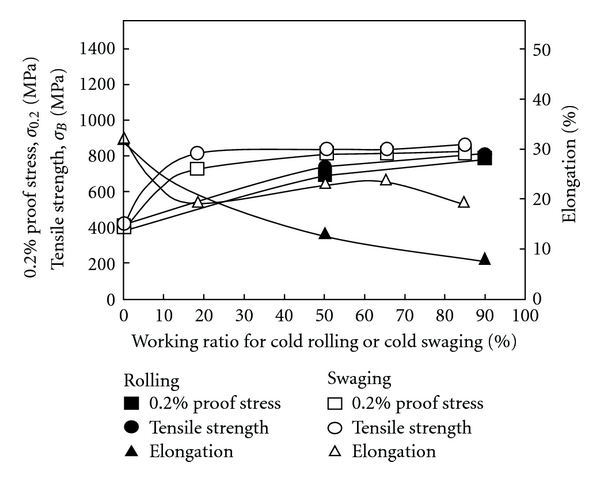
Tensile properties of TNTZ subjected to cold rolling or cold swaging as a function of working ratio.

**Figure 2:**
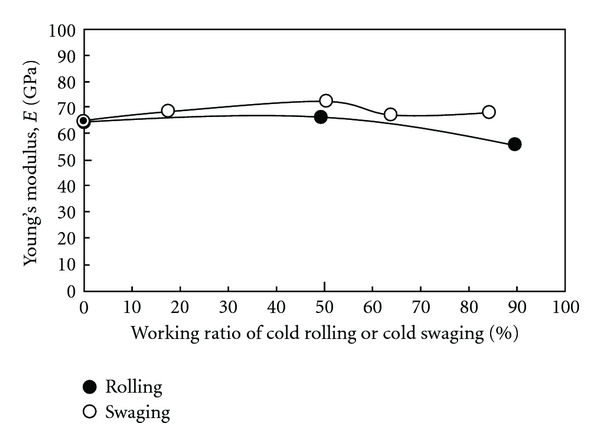
Young's modulus of TNTZ subjected to cold rolling or cold swaging as a function of working ratio.

Figure 1 [8] shows the relationships between the tensile properties and working ratio of TNTZ subjected to cold working by general cold rolling or swaging. The relationships between Young's modulus and working ratio of TNTZ subjected to severe cold working by general severe cold rolling or swaging are shown in Figure 2 [8]. The tensile strength and 0.2% proof stress increase with an increase in the working ratio and become almost equal to those of Ti-6Al-4V ELI (having tensile strength of around 800 MPa) with good elongation of TNTZ subjected to both cold rolling and cold swaging. Young's modulus of TNTZ subjected to cold rolling or cold swaging is almost constant with increasing working ratio. Young's modulus of TNTZ subjected to cold rolling tends to decrease when the working ratio is high because the trend of the formation of the texture becomes significant. 

**Figure 3:**
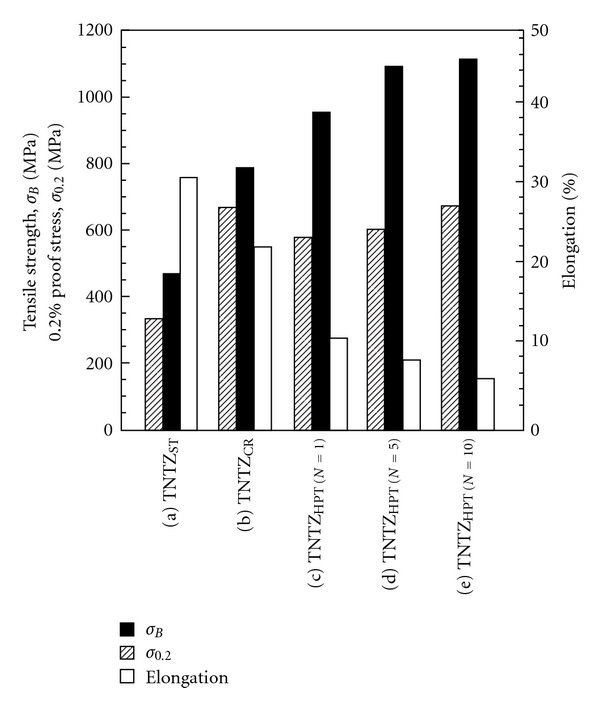
Tensile properties of TNTZ subjected to (a) solution treatment (TNTZ_ST_), (b) severe cold rolling (TNTZ_CR_), (c) HPT at a rotation number, *N*, of 1 (TNTZ_HPT(*N*=1)_)), (d) HPT at a rotation number, *N*, of 5 (TNTZ_HPT(*N*=5)_), and (e) HPT at a rotation number, *N*, of 10 (TNTZ_HPT(*N*=10)_).

Figure 3 [10] shows the tensile properties of TNTZ subjected to HPT as a function of the number of rotations, *N*. It also shows the tensile properties of TNTZ subjected to solution treatment and severe cold rolling. The tensile strength under HPT increases significantly with an increase in the number of rotations, in contrast, the elongation decreases with an increase in the number of rotations. Young's modulus of TNTZ subjected to HPT is almost constant with increasing the number of rotations although it decreases a little with increasing the rotation number. 

## 3. Improvement of Dynamic Strength while Maintaining Low Young's Modulus

**Figure 4:**
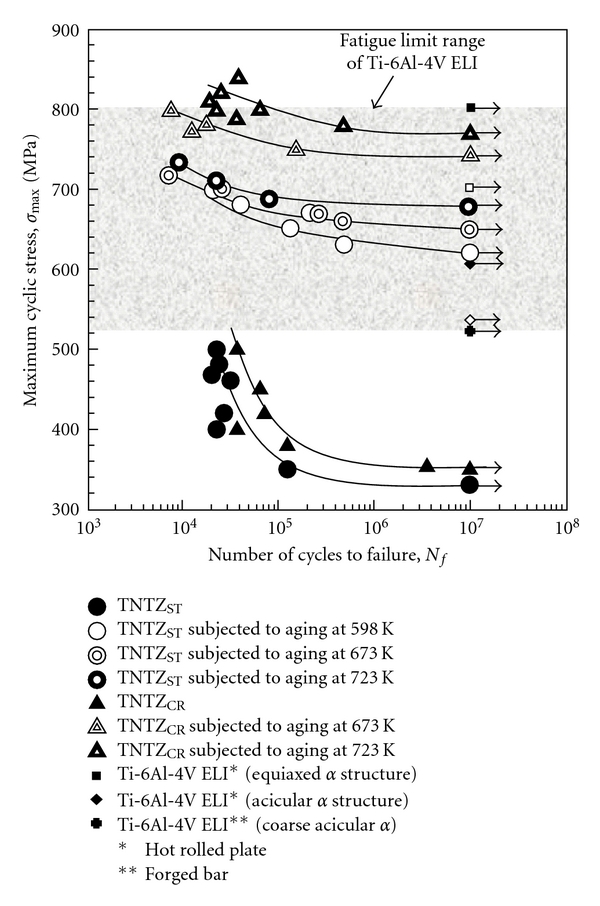
S-N curves of Ti-29Nb-13Ta-4.6Zr (TNTZ) subjected to solution treatment (TNTZ_ST_), aging at 598 K for 259.2 ks after solution treatment, aging at 673 K for 259.2 ks after solution treatment, aging at 723 K for 259.2 ks after solution treatment, cold rolling (TNTZ_CR_), aging at 673 K for 259.2 ks after cold rolling, and aging at 723 K for 259.2 ks after cold rolling along with the fatigue limit range of Ti-6Al-4V ELI.

**Figure 5:**
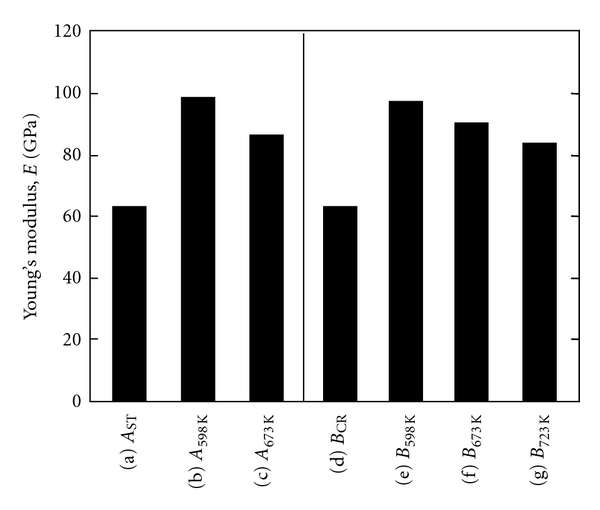
Young's moduli of elasticity of Ti-29Nb-13Ta-4.6Zr subjected to (a) solution treatment (ST) at 1063 K for 3.6 ks, A_ST_, (b) aging at 598 K for 259.2 ks after ST, A_598K_, (c) aging at 673 K for 259.2 ks after ST, A_673K_, (d) cold rolling (CR), B_CR_, (e) aging at 598 K for 259.2 ks after CR, B_598K_, (f) aging at 673 K for 259.2 ks after CR, B_673K_, and (g) aging at 723 K for 259.2 ks after CR, B_723K_.

As shown in Figure 4 [11], the dynamic strength, that is, fatigue strength of severe cold worked TNTZ is not high as compared to that of TNTZ subjected to solution treatment. The fatigue strength is significantly improved by conducting aging treatment after solution treatment or thermomechanical processing including severe cold working and aging treatment. The *α* phase or the *ω* phase precipitates in the *β* matrix phase by aging treatment. Therefore, the fatigue strength can be significantly improved by precipitation strengthening caused by the precipitation of the *α* or *ω* phase. However, as seen in Figure 5 [11], Young's modulus increases by the precipitation of the *α* or *ω* phase because Young's moduli of these phases are much higher than that of the *β* matrix phase. 

**Figure 6:**
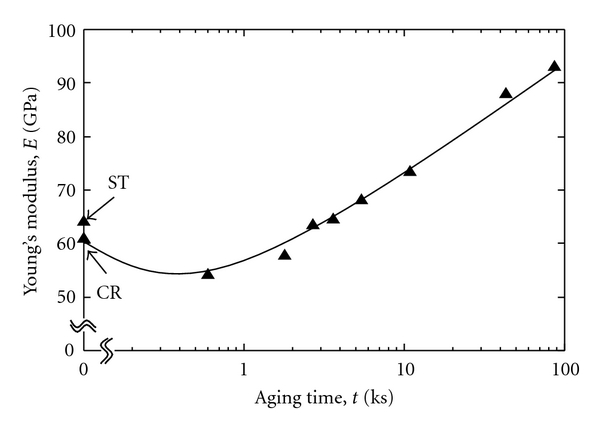
Young's moduli of ST, CR, and AT samples as a function of aging time.

**Figure 7:**
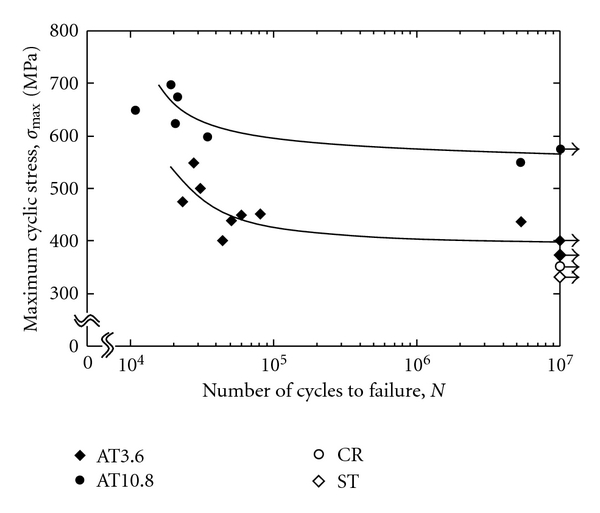
Fatigue properties of AT3.6, AT10.8 ST, and CR samples.

The *ω* phase precipitation significantly increases the strength and Young's modulus of the alloy as compared to the *α* phase precipitation although the *ω* phase enhances its brittleness. Therefore, a small amount of *ω* phase precipitation is expected to improve the fatigue strength of TNTZ while maintaining its Young's modulus fairly low. For this purpose, short-time aging at a fairly low temperature, which enhances *ω* phase precipitation by a small amount, is effective.  Figure 6 [9] shows Young's moduli of TNTZ subjected to solution treatment (ST), severe cold rolling (CR), and aging after solution treatment at a temperature of 573 K(AT) as a function of the aging time. Up to an aging time of around 10.8 ks, Young's modulus is below 80 GPa, which is a tentative target value for low Young's modulus.  Figure 7 [9] shows fatigue properties (S-N curves) of TNTZ subjected to solution treatment (ST), severe cold rolling (CR), and aging for 3.6 ks (AT3.6) and 10.8 ks (AT10.8) at a temperature of 573 K. The fatigue strength of TNTZ is improved by aging treatment for 10.8 ks (AT10.8), whereas Young's modulus is lower than 80 GPa. The TEM micrograph of AT10.8 shows the *ω* phase distribution. Thus, employing the proper precipitation method for the *ω* phase, that is, short-time aging at a relatively low temperature improves the fatigue strength of TNTZ while keeping Young's modulus low. 

**Figure 8:**
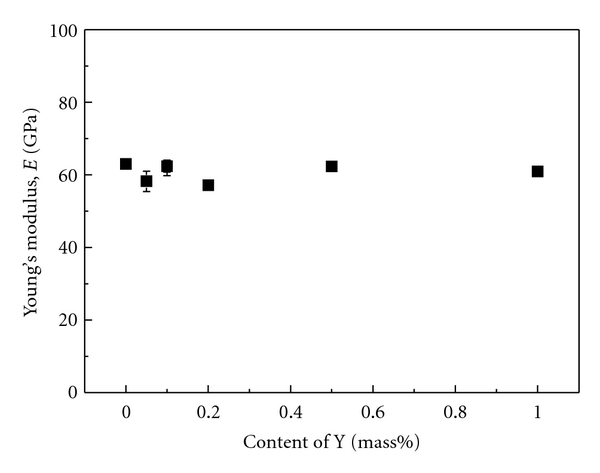
Young's modulus as a function of Y concentration in TNTZ-Y_CR_.

**Figure 9:**
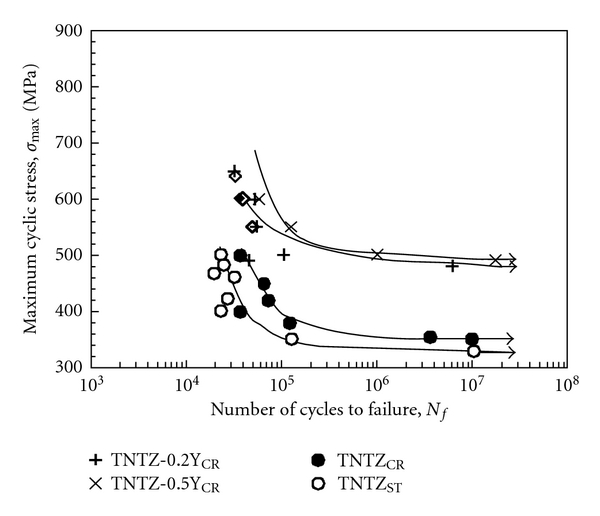
S-N curves of TNTZ with Y_2_O_3_ additions subjected to cold rolling after solution treatment along with those of TNTZ subjected to solution treatment or cold rolling after solution treatment.

**Figure 10:**
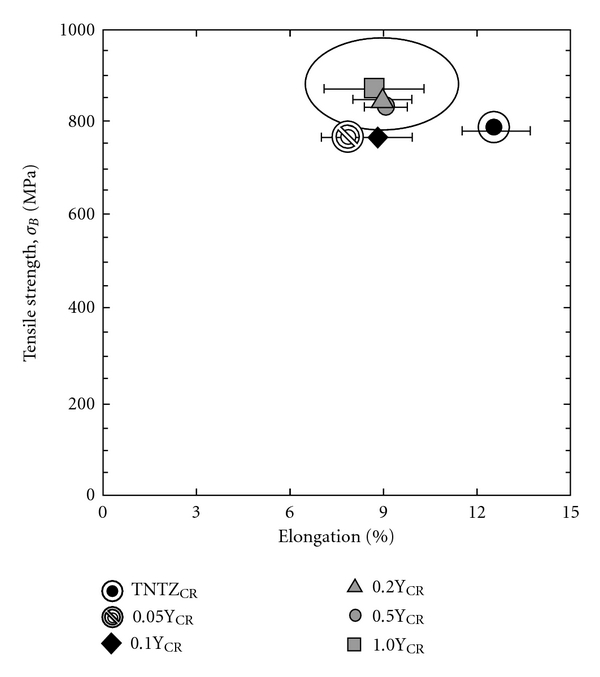
Relationship between tensile strength and elongation of TNTZ added with Y_2_O_3_ (0.05Y_CR_: TNTZ-0.05Y_CR_, 0.1Y_CR_: TNTZ-0.1Y_CR_, 0.2Y_CR_: TNTZ-0.2Y_CR_, 0.5Y_CR_: TNTZ-0.5Y_CR_, and 1.0Y_CR_: TNTZ-1.0Y_CR_) and TNTZ subjected to severe cold rolling after solution treatment (TNTZ_CR_).

The addition of a small amount of ceramic particles in the matrix is also expected to improve the fatigue strength of *β*-type titanium alloys while maintaining low Young's modulus.  Figure 8 [12] shows Young's modulus of TNTZ with Y_2_O_3_ additions subjected to severe cold rolling as a function of Y concentration. Young's modulus is nearly constant at around 60 GPa with increasing Y concentration.  Figure 9 [12] shows the S-N curves of TNTZ with 0.2 mass% and 0.5 mass% (TNTZ-0.2Y_CR_ and 0.5Y_CR_, resp.) subjected to severe cold rolling after solution treatment and the S-N curves of TNTZ subjected to solution treatment or cold rolling after solution treatment. The fatigue strength of TNTZ is improved with Y_2_O_3_ additions. Relationships between tensile strength and elongation of TNTZ added with different amounts of Y_2_O_3_ subjected to severe cold rolling (0.05Y_CR_: TNTZ-0.05Y_CR_, 0.1Y_CR_: TNTZ-0.1Y_CR_, 0.2Y_CR_: TNTZ-0.2Y_CR_, 0.5Y_CR_: TNTZ-0.5Y_CR_, and 1.0Y_CR_: TNTZ-1.0Y_CR_) and TNTZ subjected to severe cold rolling after solution treatment (TNT_CR_) are shown in Figure 10 [12]. The balance of the tensile strength and elongation of TNTZ with Y_2_O_3_ additions is excellent. 

## 4. Titanium Alloy with Self-Adjustable Young's Modulus

While using low-modulus titanium alloys, some surgeons specializing in spinal diseases, such as scoliosis, spondylolisthesis, and spine fracture, pointed out that the amount of spring back in the implant rods should be small so that the implant offers better handling ability during surgeries. The implant rods undergo bending when they are manually handled by surgeons within the small space inside the patient's body for in situ spine contouring. It is considered that the amount of spring back depends on both the strength and Young's modulus of the implant rod. If two implant rods having the same strength but with different Young's moduli are used, the implant rod having lower Young's modulus shows greater spring back. Implant rods made of low modulus titanium alloys exhibit lower Young's modulus, resulting in greater spring back. Thus, low Young's modulus, which is one of the key features of *β*-type titanium alloys such as TNTZ as a metallic biomaterial, is obviously a desirable property for patients but becomes an undesirable property for surgeons. Titanium alloys, which satisfy the requirements of both surgeons and patients with regard to Young's modulus of the implant rod, are currently being developed [13]. 

**Figure 11:**
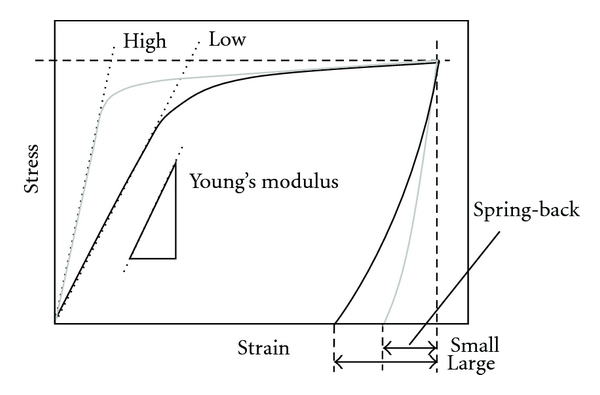
Schematic explanation of relationship between Young's modulus and spring back.

The amount of spring back is considered to be small for the alloy having higher Young's modulus as compared to that of one having low-Young's modulus as schematically shown in Figure 11. Therefore, the low-Young's modulus *β*-type titanium alloy, whose Young's modulus varies and becomes high only at the deformed part, is considered to reduce spring back and satisfy the low-Young's modulus condition as well. This concept is called self-adjustment of Young's modulus. In general, the Young's modulus of metals and alloys does not drastically change by deformation. However, in the case of certain metastable *β*-type titanium alloys, nonequilibrium phases such as *α*′,*α*′′, and *ω* phases appear in the *β* matrix during deformation [14]. If Young's modulus of the deformation-induced phase is higher than that of the original *β* phase, Young's modulus of only the deformed part of the implant rod increases, whereas that of the nondeformed part remains low. In orthopedic operations performed for the treatment of spinal diseases, the implant rod is bent by the surgeons so that it corresponds to the curvature of the spine. Therefore, if a suitable titanium alloy is employed as the implant rod material, spring back can be suppressed by the deformation-induced phase transformation that occurs during bending in the course of operation, while low Young's modulus can be retained for patients. In general, Young's modulus of *ω* phase is much greater than those of the *α*, *α*′, *α*′′, and *β* phase. Among these phases, the *ω* , *α*′, and *α*′′phase can be induced by deformation in *β*-type titanium alloys with certain chemical compositions. 

**Figure 12:**
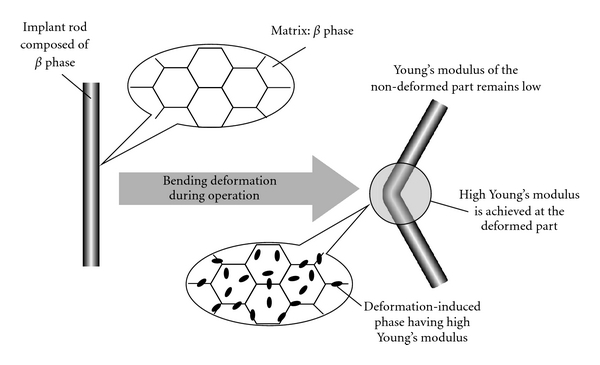
Concept of self-adjustment of Young's modulus in implant rod. If Young's modulus of the deformation-induced phase is higher than that of the matrix, Young's modulus of only the deformed part is increased by bending during operation, whereas that of the nondeformed part remains low; thus, the requirements of both surgeons and patients can be satisfied. High Young's modulus suppresses the spring back of the implant rod, whereas low Young's modulus inhibits the stress shielding effect, thereby enabling good handling ability of implant rod and remodelling of healthy bone, respectively.

One of the candidate alloys with self-adjustable Young's modulus for biomedical applications has been reported to be Ti-12Cr.  Figure 12 [13] shows Young's moduli of Ti-12Cr subjected to solution treatment (Ti-12Cr-ST) and severe cold rolling (Ti-12Cr-CR) along with those of TNTZ subjected to solution treatment (TNTZ-ST) and severe cold rolling (TNTZ-CR). Ti-12Cr-ST exhibits low Young's modulus of <70 GPa; this value is comparable to that of TNTZ-ST, which has been developed as a biomedical *β*-type titanium alloy having low Young's modulus. TNTZ-CR also shows low Young's modulus almost equal to that of TNTZ-ST. Thus, cold rolling leads to negligible change in Young's modulus of TNTZ. However, in the case of Ti-12Cr, Young's modulus increases by cold rolling and that of Ti-12Cr-CR is >80 GPa. The deformation-induced *ω* phase was detected in Ti-12Cr, but no induced phase was detected in TNTZ. Therefore, the increase in Young's modulus of Ti-12Cr is probably the deformation-induced *ω* phase transformation. 

**Figure 13:**
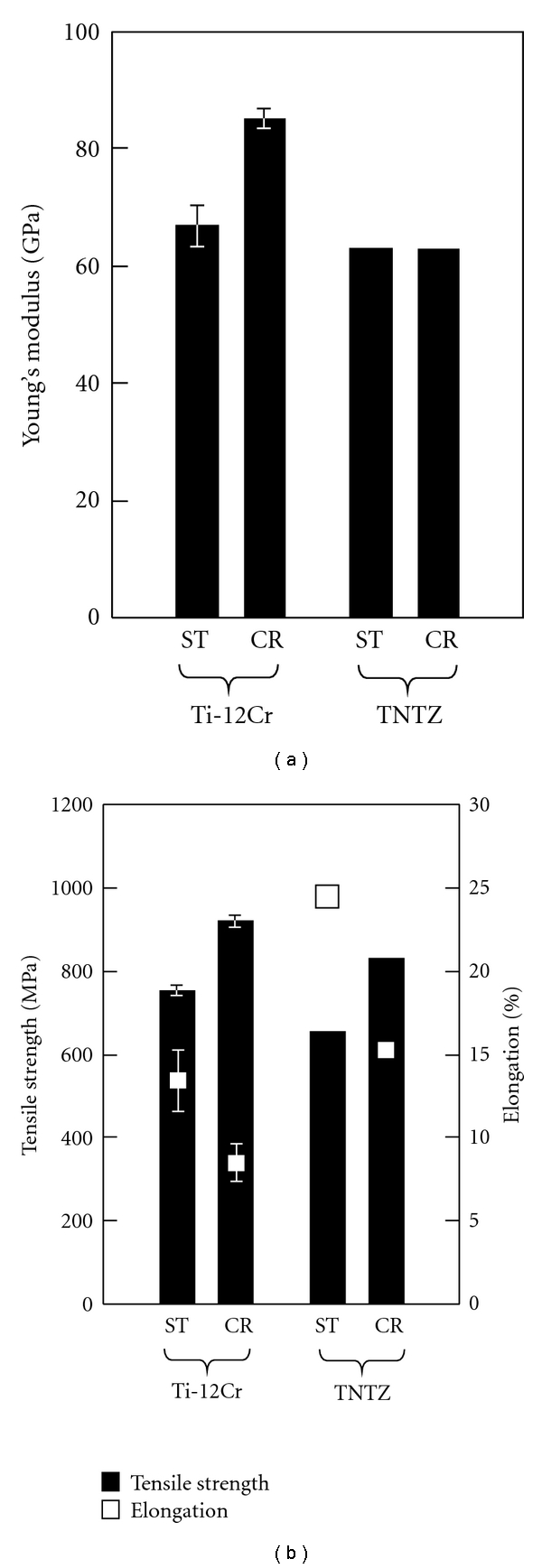
Comparison of mechanical properties of the designed alloy (Ti-12Cr) and those of an alloy with low Young's modulus (TNTZ). (a) Young's moduli of Ti-12Cr-ST, Ti-12Cr-CR, TNTZ-ST, and TNTZ-CR. (b) Tensile strength and elongation of Ti-12Cr-ST, Ti-12Cr-CR, TNTZ-ST, and TNTZ-CR. Young's modulus of Ti-12Cr clearly increases on account of cold rolling, but that of TNTZ shows almost no change. Ti-12Cr exhibits a relatively high tensile strength and acceptable elongation in comparison with TNTZ.

Figure 13 [13] shows the tensile properties of Ti-12Cr-ST, Ti-12Cr-CR, TNTZ-ST, and TNTZ-CR. The tensile strengths of both Ti-12Cr-ST and TNTZ-ST show an increase, but the elongation due to cold rolling tends to decrease. This trend is probably caused due to by occurrence of work hardening. Further, the tensile strengths of Ti-12Cr-ST and Ti-12Cr-CR can be higher than those of TNTZ-ST and TNTZ-CR, respectively. Moreover, the elongations of Ti-12Cr-ST and Ti-12Cr-CR are >10% and ~10%, respectively. High strength is an essential requirement from the viewpoint of practical application, although such high strength could lead to undesirable spring back. Therefore, the fundamental composition of Ti-12Cr makes it one of the preferred candidates for use in spinal fixation devices as a biomedical titanium alloy with the ability to self-adjust its Young's modulus. 

## 5. Low-Young's Modulus Titanium Alloys for Use in Removable Implants

In the case of some types of internal fixation devices implanted into the bone marrow such as femoral, tibia, and humeral marrow, in the case of screws used for bone plate fixation [15], and in the case of implants used for children, which otherwise would grow into the bone, it is essential to remove the internal fixation device after surgery owing to certain specific indications; these indications include significant local symptoms such as palpable hardware, wound dehiscence/exposure of hardware, or athletes returning to contact sport [16, 17]. The assimilation of removable internal fixation device with the bone due to precipitation of calcium phosphate might cause refracture of the bone during the removal of the fixation device. Therefore, in these cases, it is essential to prevent the adhesion of the alloys with the bone tissues. Hence, considering this requirement, it is essential to inhibit the precipitation of calcium phosphate. It is reported that Zr, which is nontoxic and allergy-free element, has the ability to prevent precipitation of calcium phosphate [18] and Ti alloys with Zr contents exceeding 25 mass% prevent the formation of calcium phosphate, which is the main component of human bones [19]. Thus, Ti-30Zr-Mo has been proposed as low Young's modulus titanium base biomaterials for use in removable implants.

**Figure 14:**
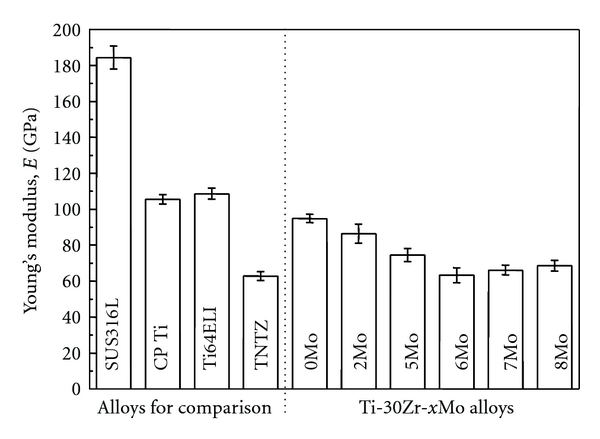
Young's moduli of Ti-30Zr-*x*Mo (*x* = 0, 2, 5, 6, 7, and 8 mass%) alloys subjected to solution treatment and the alloys considered for comparison.

Figure 14 [20] shows Young's moduli of Ti-30Zr-*x*Mo alloys subjected to solution treatment and those of the alloys considered for comparison. Young's modulus of Ti-30Zr-*x*Mo is lower than that of the alloys considered for comparison except TNTZ. Young's modulus of 6Mo shows a minimum value of around 60 GPa, and TNTZ also shows low Young's modulus. 

**Figure 15:**
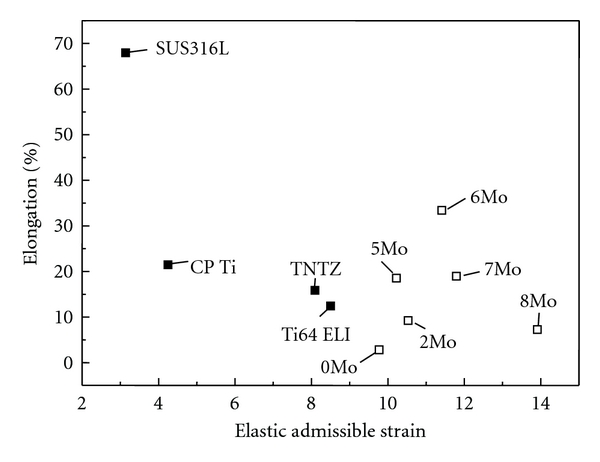
Distribution of solutionized Ti-30Zr-*x*Mo (*x* = 0, 2, 5, 6, 7, and 8 mass%) alloys and the alloys considered for comparison in a plot of elastic admissible strain against elongation.

**Figure 16:**
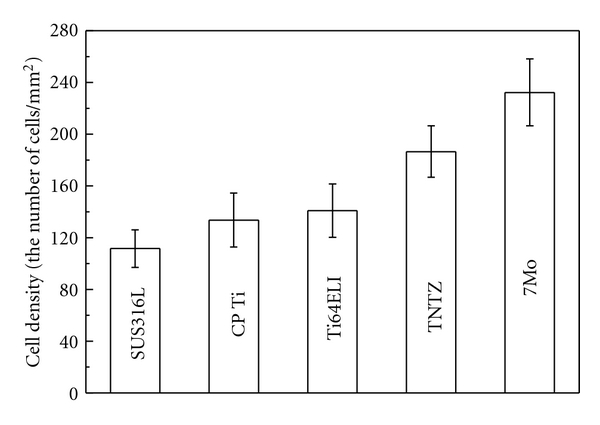
Density of cells cultured in 7Mo and the alloys considered for comparison for 24 h.

In orthopedic applications, ideal biomedical implant materials are required to have high strength and low Young's modulus. The elastic admissible strain, defined as the strength-to-modulus ratio, is a useful parameter considered in orthopedic applications. The higher the elastic admissible strain is, the more suitable are the materials for such applications [21].  Figure 15 [20] shows the distribution of the as-solutionized Ti-30Zr-*x*Mo alloys and the alloys considered for comparison in the plot of elastic admissible strain against elongation. 6Mo and 7Mo exhibit larger elongation and higher elastic admissible strain than those of the other Ti-30Zr-*x*Mo and SUS316L, CP Ti, Ti64 ELI, and TNTZ.  Figure 16 [20] shows the density of the cell cultured in 7Mo and the alloys considered for comparison, for 24 h. 7Mo has the highest value of cell density. Therefore, 6Mo and 7Mo show promising potential to be new candidates for use in biomedical applications. 

## 6. Young's Modulus and Stress Shielding

**Figure 17:**
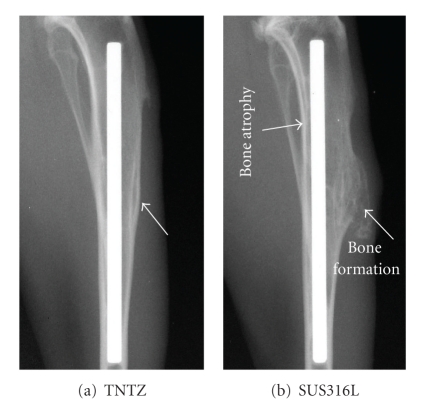
X-ray photographs of intramedullary rods made of (a) TNTZ and (b) SUS316L stainless steel at 24 weeks after implantation into tibiae of rabbits.

It is very important to prove that the implant has Young's modulus similar to that of the bone, which will inhibit bone atrophy and good bone remodeling [1, 4]. Of course, the geometry of implant is another factor to control Young's modulus. However, we focus on Young's modulus of implant in this paper. Studies have been conducted on the implantation of intramedullary rods and bone plates made of low-Young's modulus *β*-type titanium alloy (TNTZ), conventional practical (*α* + *β*)-type titanium alloy (Ti-6Al-4V ELI), and conventional stainless steel (SUS316L) into fracture models made into the tibiae of rabbits [8, 22]. Young's moduli of TNTZ, Ti-6Al-4V ELI, and SUS316L stainless steel used for intramedullary rods, which were measured by three-point bending tests, were 58, 108, and 161 GPa, respectively. In both cases, the implantation of intramedullary rod and the implantation of bone plate, bone atrophy, and bone remodeling have been reported to be the least and the best, respectively, for TNTZ.  Figure 17 [8, 22] shows the X-ray photographs of the fracture models implanted with intramedullary rods made of TNTZ and SUS316L stainless steel at 24 weeks after implantation. Bone atrophy can be observed at the upper rear portion of the tibia for the intramedullary rod made of SUS316L stainless steel, but no bone atrophy is seen for the intramedullary rod made of TNTZ. Large bone formation can be observed at the front part of the tibia for the intramedullary rod made of SUS316L stainless steel, but very small bone formation can be observed at the front part of the tibia for the intramedullary rod made of TNTZ. Therefore, low Young's modulus is effective in inhibiting bone atrophy and leads to excellent bone remodeling. 

**Figure 18:**
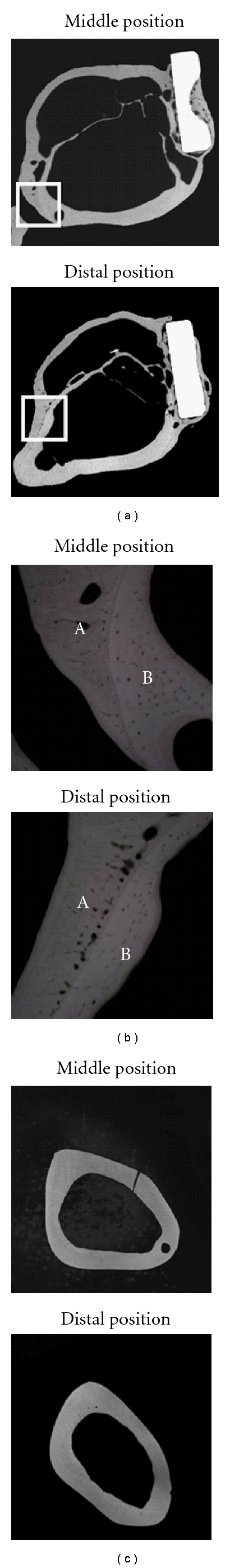
CMRs of cross-sections of fracture models implanted with and without bone plates made of TNTZ at middle position and distal position at 48 weeks after implantation: (a) cross-section of fracture model, (b) parts of □ of (a), namely, high magnification CMR of branched parts of bones formed outer and inner sides of tibiae, and (c) cross sections of unimplanted tibiae.

Further study on the effect of Young's modulus on the bone remodeling has been done by implanting bone plates made of TNTZ, Ti-6Al-4V ELI, and SUS316L stainless steel into the fracture models made in tibiae of rabbits. Only for the case of the bone plate made of TNTZ, the increase in the diameter of the tibia and the double-wall structure in the intramedullary bone tissue has been reported to be observed as shown in Figure 18 [1]. For Figure 18, the inner wall bone structure is the original cortical bone, namely, the remained old cortical bone and the outer wall bone structure is newly formed one. This is the possible result of the bone remodeling with the low-Young's modulus bone plate. 

**Figure 19:**
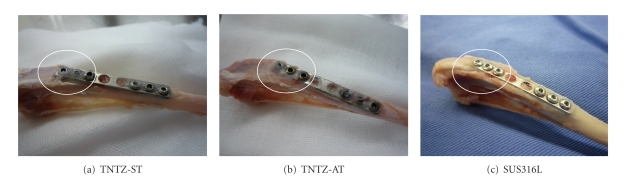
Profiles of extracted bone plates made of (a) TNTZ-ST, (b) TNTZ-AT, and (c) SUS316L stainless steel fixed to tibiae of rabbits at 52 weeks after implantation.

Furthermore, it is necessary to understand the level of Young's modulus, which is effective for inhibiting bone atrophy and enhancing bone remodeling.  Figure 19 [8] shows the profiles of the extracted bone plates made of TNTZ subjected to solution treatment (TNTZ-ST), TNTZ subjected to aging after solution treatment (TNTZ-AT), and SUS316 L stainless steel (SUS316L) attached to the tibiae of rabbits at 52 weeks after implantation. Young's moduli of TNTZ-ST, TNTZ-AT, and SUS316L, which were measured by three-point bending tests, were 58, 78, and 161 GPa, respectively. The upper and side surfaces of each bone plate made are covered by newly formed bone, but a fairly large amount of newly formed bone can also be observed on the heads of the screws made of TNTZ-ST and TNTZ-AT, which are surrounded by circle marks. 

**Figure 20:**
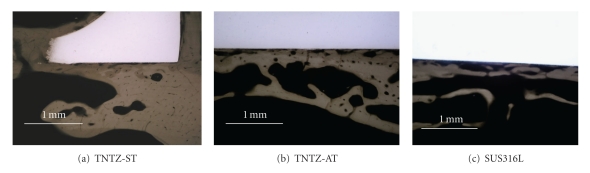
Optical photographs of bones formed around extracted bone plates made of (a) TNTZ-ST, (b) TNTZ-AT, and (c) SUS316L stainless steel fixed to tibiae of rabbits at 52 weeks after implantation.

Figure 20 [8] shows the optical micrographs of the bone state beneath the bone plates made of TNTZ-ST, TNTZ-AT, and SUS316L. Bone atrophy can be observed in all cases but becomes more distinct with increasing Young's modulus; it is the highest for SUS316L, followed by TNTZ-AT and TNTZ-ST. It is expected that titanium alloy with even lower Young's modulus will be advantageous in inhibiting bone atrophy and lead to much better bone remodeling. 

## 7. Summary

Titanium base alloys with low Young's modulus have been proved to be effective for inhibiting bone atrophy and enhancing bone remodeling by conducting animal tests in rabbits. Therefore, low Young's modulus titanium alloys are expected to be useful in practical applications such as implant devices used for replacing failed hard tissue. However, considering the ease of the operation, other properties such as small spring back, which is effective in maintaining the bending shape of the implant in the body, and low yielding stress and high ultimate strength, which lead to ease in achieving permanent deformation of the implant in the narrow space in the body, are also important. Metallic biomaterials, which satisfy the demands of both patients and surgeons, are highly required.
